# Hepatitis C Diagnosis and Treatment Among Indigenous People in a Canadian Context: Challenges and Community-Led Solutions

**DOI:** 10.3390/microorganisms12112364

**Published:** 2024-11-19

**Authors:** Kate P. R. Dunn, Mia J. Biondi, Samuel S. Lee

**Affiliations:** 1 School of Nursing, York University, Toronto, ON M3J 1P3, Canada; 2 Viral Hepatitis Care Network, University Health Network, Toronto, ON M5G 2C4, Canada; 3 Cumming School of Medicine, University of Calgary, Calgary, AB T2N 4N1, Canada

**Keywords:** hepatitis C, care and treatment, interventions, Indigenous-led, community-engagement

## Abstract

The historical and ongoing impacts of the influence of colonization are experienced by Indigenous people in systemic racism, inequity in healthcare access, and intergenerational trauma; originating in the disruption of a way of life and seen in a grief response, with links to disparate hepatitis C virus (HCV) prevalence. Despite this, the focus often remains on the increased incidence without a strengths-based lens. Although HCV is a global concern that can result in cirrhosis, liver failure, or cancer, diagnosing and linking people to care and treatment early can prevent advanced liver disease. Efforts to engage certain priority populations are occurring; however, historical context and current practices are often forgotten or overlooked. This is especially true with respect to Indigenous people in Canada. This review considers the published literature to elucidate the context of historical and ongoing colonizing impacts seen in the current HCV treatment gaps experienced by Indigenous people in Canada. In addition, we highlight strengths-based and Indigenous-led initiatives and programming that inspire hopefulness and steps toward community-engaged solutions to meet the World Health Organization Goals of eliminating HCV as a public health threat.

## 1. Hepatitis C and Efforts Towards Elimination as a Public Health Threat

The historical and current context leading to the disproportionate experience of hepatitis C virus (HCV) with Indigenous people in Canada reflects the research literature. This research often highlights Indigenous populations in Canada by pointing to high mortality rates, a focus on disparity, and a narrative that leaves Indigenous people in the disparity spotlight, with the impression that most people who use drugs or people who are incarcerated are Indigenous, and that being a priority population is a detriment. Conversely, the ongoing impacts of colonial disruption on wellness are integral considerations in the HCV context.

HCV is a virus transmitted through blood-to-blood contact, leading to inflammation of the liver which may result in cirrhosis, liver cancer, or liver failure. The current treatment consists of oral medication for eight or twelve weeks, resulting in a sustained viral response of over 95%, or cure. The World Health Organization (WHO) has identified viral hepatitis as the world’s second-leading fatal infectious disease, with rising rates and 3500 deaths per day [1]. Considering these numbers, a strategy and timeline has been recommended by the WHO to eliminate HCV as a public health threat by the year 2030, reducing new infections by 80%, diagnosing 90% of people who have HCV, and initiating treatment with 80% of those living with current HCV infections [2,3].

In 2016, Canada made a commitment to align with the WHO global elimination goals. At the national level, the *Blueprint to Inform Hepatitis C Elimination Efforts in Canada*, serves as guidance toward action in various settings across Canada and prioritizes those impacted most by viral hepatitis. This blueprint identifies six priority populations: people who are incarcerated; people who use drugs (PWUD); Indigenous people (First Nation, Métis, and Inuit); gay, bisexual, and other men who have sex with men (gbMSM); newcomer and immigrant people from countries with high prevalence rates; and people born between 1945 and 1975 [3].

In this context, Indigenous people are the only priority group identified because of who they are versus an action or circumstance and are also represented in all but one of the other prioritized groups. Fayed et al. illustrates the multi-point importance of this misclassification by highlighting the distorted universality of HCV risk between populations and the visual comparison of representation in high-risk groups [4]. Instead, they propose a reconciliatory-sited orientation, which is Indigenous-centered, recognizing that colonialism has contributed to the overrepresentation of Indigenous people in Canada among, and hence at the center of, populations that are at high risk for HCV.

When these priority population classifications align, multiplied inequity, as seen in the research, shows that the lowest probability of a person accessing curative medication for HCV is associated with being Indigenous, being someone who injects drugs, and identifying as a woman [5]. As Lee et al. note “Indigeneity is erroneously portrayed as a risk factor, failing to underscore the historical and contemporary inequities that are actually responsible for such over-representation”, while also deprioritizing and devaluing the need for Indigenous-led initiatives [6].

Research has shown Indigenous people respond to direct acting antiviral (DAA) therapy in similar rates to the wider population, and possibly have higher success with self-clearance of HCV [7,8]. But the variation in HCV prevalence in Indigenous communities is four to eleven times higher, reflecting the considerable disparity of available data, the varied access to screening and care across Canada, and the disproportionate representation in the other priority groups [4,5,9,10,11,12,13,14,15,16,17].

The historical and ongoing impacts of colonial disruption on wellness are integral considerations in this context, leading to the disproportionate experience of HCV with Indigenous people in Canada. Conversely, Indigenous ethnic diversity across nations, Indigenous culture, traditional wholistic wellness perspectives, and community connections are a protective strength of Indigenous identity with direct links to liver wellness.

## 2. Connections Between Colonial Context and HCV in Current Experience

HCV was identified in 1989 [18], but its connection to history goes back much further for Indigenous people, in what is now called Canada. The First People living in Canada prior to colonization comprised numerous First Nations with distinct cultures, languages, and governance practices. Today, the term Indigenous people is inclusive of three distinct people and culture groups: First Nation people from numerous tribes and regions across Canada; Inuit of the vast Arctic and sub-Arctic lands; and Métis, who are descendants of unions between French Europeans and First Nation people.

The history of these people is connected to the land and is rich with creation stories, traditions connected to geographical points of interest, and the stories of ceremonies, battles, winter counts, natural wonders and migrations; long before documented dates. Following European explorers’ arrival in what is now Canada, the settlement of the vast wild country followed patterns of other colonized countries, where the goal was to control Indigenous people by conversion to European religion, relocation to remote reserved lands now called reserves, and disruption of all aspects of life, including spirituality, rituals, subsistence, governance, education or knowledge transfer, language, lifestyle, child-rearing, diet, housing, clothing, and wellness practices.

Legislation including the Indian Act of 1876 continues today to define the relationship between the government, Indigenous people, and health outcomes. The process of colonization in Canada rationalized the drastic disruptions to Indigenous wellness and the trauma experienced by generations of Indigenous people. Purposeful disruptions include the imposed system of elected chief and council to displace and control inherent authority and governance by separating the wisdom of elders, spiritual leaders, healers, and women from collective voice. Disruptions to the fabric of a wellness-based society also included denying the rights of Indigenous women and their children if marrying anyone outside their own band; and confining First Nation people to reserved land, curtailing wellness-supporting activities, such as traditional food subsistence patterns, cyclical gatherings for spiritual practice, medicinal plant gathering, trade and inter-tribal marriage, or social milestone events.

Disruptions to wellness practices are further seen in legislative action with intent to isolate and disconnect Indigenous people from culture and community through forced attendance in residential schools, by being sent to tuberculosis sanitariums, or through continued experience of racism in the education system, justice system, or healthcare system. These forced displacements and disruptions to a wellness-based life are carried in blood-memory impacts seen today in visible and invisible health outcomes. As Fayed et al. illustrate, the collective loss and cumulative ethno-stress of historic and ongoing trauma leads to a psycho-emotional response from unresolved grief, and may be seen as “emotional deregulation, and psychological challenges, including substance use” [4].

Substance use is complex and is the result of numerous intersecting factors, including access and equity in healthcare and housing, and is often aligned with race instead of being more appropriately linked to the multiple disruptive factors, such as social, structural, and political determinants of health. Although substance use may mask unresolved grief from generations of disruption, it also carries a burden of stigma and accompanying continued spirals of emotional deregulation, psychological challenges, and continuing intergenerational patterns. Infectious diseases, including HCV, compound the stigma and exacerbate self-stigma related to identity as an Indigenous person, to using substances, and to the risk of, or transmission of, an infectious disease [19]. HCV is a blood-borne virus which only requires one drop of blood for transmission to occur, which means transmission can occur through sharing a razor, toothbrush, or nail-clippers. However, the highest risk and most common mode of transmission is through shared injection supplies for substance use, thus adding to the stigma and grief burden.

Although Indigenous people are disproportionately impacted by HCV, there is a gap in the literature showing inclusion of an Indigenous perspective in HCV initiatives, funding, and programming. Most articles briefly mention Indigenous peoples as a priority population. Many describe data supporting the increased prevalence of HCV by Indigenous peoples; several link the current experience of high HCV rates as a colonial legacy; but only a few illustrate models of care, connection to culture, or wellness-based initiatives seeking healing or redirection of the current disparaging storyline.

The evolution of diagnoses and treatment of HCV has increasingly become simplified and accessible for the general urban population. While testing previously involved a complicated, expensive, and specialty-controlled care algorithm requiring multiple laboratory visits and diagnostic steps, this has been greatly simplified, including the use of point-of-care testing. In addition, treatment is all-oral and extremely well tolerated, with high cure rates [20]. Despite these advancements, disparity and inequity still occur, due to limited infrastructure and funding, as well as care models designed for the urban context [21,22]. An example of an experience in differing settings is portrayed by recent retrospective First Nation administrative data analysis in select regions in the province of Ontario, Canada, where it took 288 days to follow up initial HCV antibody testing with next-step labs when living in an off-reserve community, versus 68 days when living in an on-reserve community [23]. This was followed by the differential of people who continued the process and accessed HCV treatment, where 43.7% lived off-reserve and 33.7% lived in a reserve community. The literature identifies gaps in the infrastructure and the privileged system of healthcare in Canada, where we could be doing better.

## 3. Acknowledgement of Indigenous Lived Experience with Current HCV Approaches

The intergenerational and ongoing impacts of experiencing colonial jurisdiction and control as an Indigenous person in health-related issues has fostered a mistrust of healthcare systems, healthcare providers, health messaging, and medical diagnostics [24]. Because HCV is identified as a colonial illness by Indigenous people, it is imperative to work within an anti-colonial awareness that supports renewal and connection to healing approaches built on a foundation of culture, wellness, and trust [4]. As is the case with most healthcare systems, testing and treatment pathways in Canada are designed with an urban setting and Caucasian patient in mind [25]. Therefore, many Indigenous patients identify loneliness, anxiety, and fear as barriers to accessing healthcare related to HCV treatment [5]. The literature highlights further gaps in culturally supportive care identified by patients, including medical dismissal by healthcare staff, racial discrimination, stigma toward history of substance use, perception of non-urgent status of HCV, lack of trauma-informed approaches, disdain for traditional healers and traditions, linear and rigid timelines, stark clinic spaces, disinterest in relational conversation or engagement, disease versus wellness approach, and exclusive-to-individual instead of community awareness, resulting in potential disengagement or delay in care [4,9,12,16,26,27,28,29].

## 4. Community-Directed Knowledge Sharing and Integration

Knowledge is integral to all interactions related to health and wellness, and yet there is a notable gap in knowledge from multiple perspectives related to HCV. Non-stigmatizing educational resources are needed for healthcare providers, as well as priority patient populations, alleviating both reticence to treat and fear of treatment, scaling up capacity, and removing systemic barriers [9,30]. Although effective clinical education modules have been designed to fill this gap in knowledge, when healthcare providers are unaware of basic information on HCV, they are unable to provide intersectional support or non-stigmatizing care, or assess barriers to treatment, especially for populations who face systemic barriers or cultural differences [9,30].

Cultural awareness is an integral component of all harm reduction, liver disease, sexual health, plant medicine, or wellness conversations [9,31]. While most literature includes a conclusion statement calling for creation of culturally safe spaces, cultural awareness training, or culturally relevant prevention [16,27], there is little evidence that these principles are being enacted. For example, Indigenous community wellness and resilience are reliant on connections to culture, land, ceremony, traditional knowledge sharing, and community-based approaches focusing on wholistic wellbeing. This should be the focus of engagement, funding, strategy, innovation, and awareness campaigns [9,16,27,28].

## 5. Relevant Data Connected to Community Experience

The Public Health Agency of Canada implemented a community-led survey opportunity called *Tracks* from 2018 to 2020 to ascertain a snapshot of data around social determinants, harm reduction, and sexual health with Indigenous communities, accompanied by laboratory testing for HIV, HCV, and syphilis to improve understanding of structural and behavioral influences. The results further highlight the continued colonial legacy, and stigma connected to the data [13,16,32]. Retrospective First Nation administrative data analysis for several regions in Ontario showed 60% of First Nation people who completed laboratory testing and showing an active HCV infection did not engage in treatment, and for those who did, the time from testing to treatment was over 10 years [23]. Together, these data highlight the importance of pairing data with living experience [33] to understand the barriers experienced in the testing-to-treatment pathway, and how we can improve this experience through capacity, infrastructure, and policy changes. True prevalence is not well understood, as several provinces in Canada do not gather HCV prevalence data with Indigenous communities. As a result, there are challenges in shaping not only the current situation for HCV as it is experienced within reserve and off-reserve community settings, but also the rationale for funding support and evaluation for effectiveness of programming and implementation shifts in HCV screening and treatment [34,35]. Unfortunately, quantitative research continues to focus on Indigenous status as a risk factor, and the widening disparity, increasing mortality, rising HCV incidence, and lack of available data [10]. Data adds value in telling a story, but when the key storyline of intergenerational and ongoing colonial influence is left out, the story becomes skewed [4,9,13].

## 6. Wellness-Focused Equitable Health Care

The treatment pathway for HCV has limitations in capturing individual needs, as well as community needs, due to its original design for an urban setting and assumptions that people will have funds for transportation, food, and housing; with be health literate; and will be able to self-navigate the healthcare system [9,21,36,37]. For example, although DAAs are on the formulary as a covered medication benefit in Canada, there must be a connection to healthcare to access a prescriber and prescription, and accessing varied services in multiple locations, such as the laboratory, prescriber, pharmacist, or counselor, may prove to be an unsurmountable barrier for some people [9,38].

Disclosure of ethnicity, medical diagnosis, substance use, or a different lived experience of socio-economic status shifts the experience of care received, resulting in many people not being connected to HCV care or treatment [9,23]. Resulting from a lack of equitable access to primary care, many Indigenous people seek their healthcare from the emergency department, who may deprioritize HCV care, which creates further priorities for incorporating increased awareness of HCV in healthcare teams across all avenues of care, while also creating increased opportunities for HCV screening through linking accessible programming [27,35]. Gaps in care are often experienced when social instability impacts the capacity to initiate, or stay connected to, a treatment plan; when paired with a lack of communication, and a sense of devaluation and stigma; the experience may be overwhelming [5,26,28,39].

**Figure 1:**
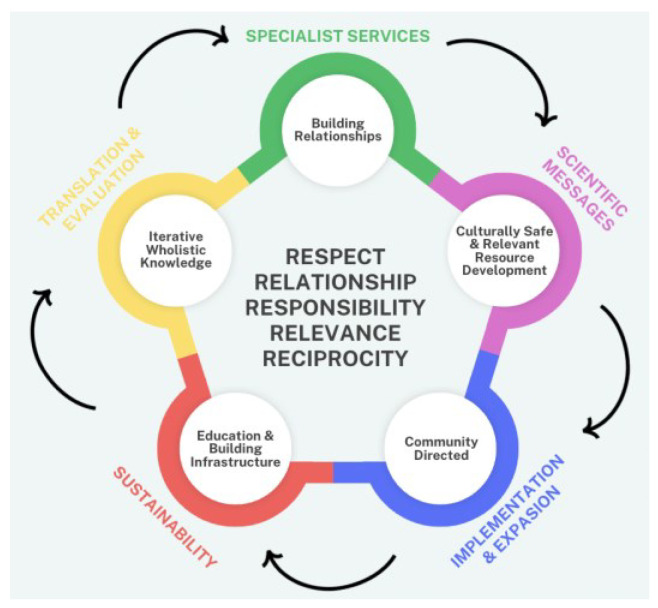
The 5 Rs of respectful engagement form the central foundation supporting the community-based framework for HCV care, as illustrated by reflecting the funder-required work-streams (outer arrows) alongside community-directed priorities, oral knowledge, and Indigenous perspectives of wellness combined with biomedical expectations of project outputs (outer circles). Adapted from [43].

Minimizing structural barriers is important, but perhaps more relevant are considerations on how to respectfully tailor specific interventions to engage Indigenous people [12,40]. The persistent inequity and cavernous gaps in Indigenous healthcare illustrate the continued disassociation between the ongoing burden of colonial harms and a disparaging lack of decolonized, culturally connected, wellness-focused, land-based, and self-governance-shaped care approaches [4,11,16,28,41]. Specific gaps were also noted in programming and specific approaches for women [5,34,39,42] and for straight men. Figure 1 describes the five Rs of respectful engagement, identified as respect, relationship, responsibility, relevance, and reciprocity, as the foundation to support community-based frameworks for collaborative and engaged HCV care.

## 7. Successes Through Micro-Elimination, Task-Shifting, and Creative Innovation

Micro-elimination of HCV is a concept that describes concerted efforts in an environment, community, region, population, or other, to focus on awareness, education, screening, and linking to treatment while consciously removing barriers. It typically involves using non-traditional or task-shifting models while engaging multi-stakeholder support. Efforts towards engagement with and for Indigenous people sharing positive impacts include micro-elimination projects, nurse-led models, telemedicine approaches, and co-created media and data models such as producing a film. Micro-elimination is often more achievable and less costly, and inspires further momentum through regional successes, as well as supporting collaboration with a variety of stakeholders to build awareness, de-stigmatize, and focus on regional projects. These projects are inclusive of other specific approaches mentioned below, and focus on treatment as prevention, treatment as harm reduction, and bringing HCV care to specific segments of the population instead of telling people to seek care [13,29,44]. A salient example in the literature is a micro-elimination effort in an urban center in British Columbia where a nurse-led model provided screening and treatment to Indigenous and non-Indigenous residents and their social connections within a specific housing site [44]. Through this initiative, 180 people who would likely not otherwise engage in healthcare were screened and, of those, 39 were supported through the treatment journey to cure. Another example of a successful endeavor was in a remote community in Saskatchewan where comprehensive engagement built on the existing *Know Your Status* program designed for HIV. Here, community-directed wholistic awareness to included steps to HCV screening and treatment, as well as knowledge translation [22]. Other examples include awareness activities, media projects, information sessions, community-wide events focused on liver health, point-of-care screening, phlebotomy for further diagnostic labs, fibroscanning, and case management through nurse-led and outreach teams. These efforts responded to community needs and thereby reduced stigma and supporting strategies for prevention, as well as engaging people directly with care [21,22,45,46]. This approach focuses supports on “self” in the community instead of only self as an individual patient, thus encompassing traditional Indigenous perspectives of wellness, culture, history, and relationality [21].

Collaboration between remote communities and urban partners supports capacity building and connections to further resource access, as highlighted by two Indigenous communities in Northern Ontario who involved an urban specialist clinic to facilitate training and implementation by collecting samples for testing using dried blood spot (DBS) cards [47]. Community-driven strategy design supported the initiative and included awareness through media, presentations with youth in the school, presentations and screening for community members, and linkage to healthcare teams and social support programming, resulting in over 700 people completing HCV screening [47].

Nursing as a profession often covers innumerable aspects of healthcare, and thus may have a trusting relationship with community members and priority populations while placing them as potential leaders in task-shifting initiatives focused on building relationships, increasing awareness, and taking healthcare and HCV screening directly to patient populations and supporting treatment as harm reduction [13,20,29,44,48]. The nursing role supports wholistic engagement with opportunities for an inclusive approach to multiple health topics versus a specific disease focus, and paired with an Indigenous health promotor and or peer support worker, provides a wrap-around support for community engagement to include Indigenous culture, ceremony, wellness perspectives, trauma, and history-informed approaches and supportive trust-building relationships [27,29,48]. Nurse-led team approaches have been shown to decrease barriers in both urban and remote settings while increasing collaboration with community stakeholders, healthcare supports, and community members to increase screening accessibility and success, as well as supported linkage to care and successful completion of treatment [48,49].

Remote communities across Canada face disparities in access to not only basic healthcare, but also to specialist care. Telemedicine or similar virtual modes of communication have increasingly provided opportunities to support HCV treatment through virtual access to hepatology and gastroenterologist specialty consults, thereby facilitating HCV care, often in partnership with nurse-led or peer-led models toward alleviating common barriers such as transportation from the remote community [6,50,51]. In Alberta, ECHO (Extensions for Community Health Outcomes) not only provides this virtual link to specialist care, but also further invests in supporting access by facilitating HCV awareness presentations for community healthcare teams and community members, co-creation of culturally connected awareness resources such as booklets, posters, social media posts and newsletters, and support of knowledge sharing events and liver health day events that include HCV screening, fibroscans, and cultural wellness presentations [43]. Virtual access programming provides options for people facing logistical barriers yet shows comparable cure success rates to those seen in traditional models of care, and in fact may increase engagement to complete treatment [50,52].

**Figure 2:**
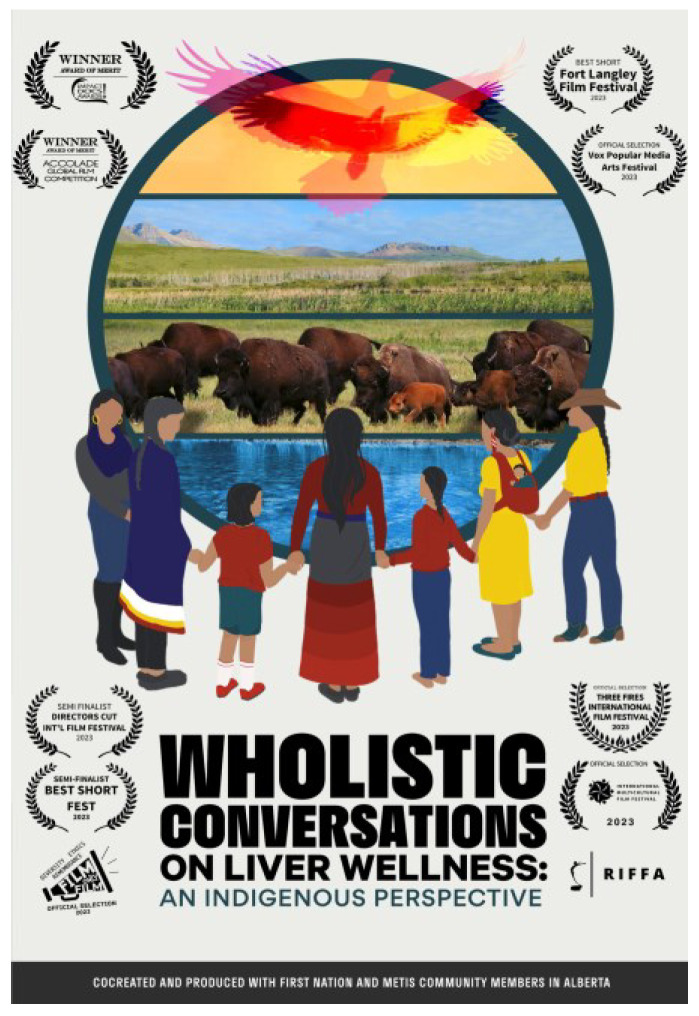
This DocuStory film [53] shares conversations on liver wellness featuring stories from personal and professional experiences and various seasons of life. These eye-opening conversations share the impact the Indigenous community is making on awareness for the importance of liver health rooted in traditional culture and ways of life. These conversations put emphasis on looking forward to teaching future generations the importance of the liver, and how imperative it is to overall health and well-being. Co-created and produced with First Nations and Métis community members in Alberta, Canada, this work stemmed from relational discussions, of which more are urgently needed to better understand the impact of interventions in a manner that does not just “count” those tested and treated. Watch the full video at: https://cumming.ucalgary.ca/resource/echo/home#liver-wellness-film (accessed on 24 October 2024).

Awareness resources on health topics typically feature written resources from a biomedical science perspective, which does not engage cultural or wholistic perspectives, and often leaves Indigenous communities unengaged and unaware. Co-created with Indigenous knowledge keepers and elders in Alberta, Canada, a DocuStory film (Figure 2) combined culture, land-based visuals, and traditional storytelling to create a knowledge sharing or knowledge translation resource on HCV prevention, harm reduction, and liver wellness perspectives, connecting not only biomedical facts around HCV but also a heart engagement with the connection to personal wellness. This approach de-stigmatizes an often-stigmatized topic, and can be shared in a wide variety of settings, fostering trust and creating a space for open discussion at community health fairs, screening events, corrections centers, clinics, workshops, and classrooms [53]. Although not intended to be a pan-Indigenous resource, there is relevance for many settings. The film format is accessible on any smart device, is freely accessed nation-wide, and has received honors in national and international film festival as an innovative culturally connected health awareness resource.

## 8. From Blueprint to Roadmaps: A Regional Response to HCV

Following the release of the *Blueprint to Inform Hepatitis C Elimination Efforts in Canada* [3], with the leadership of the Canadian Network on Hepatitis C, multiple regions began to create what are now referred to as regional *Roadmaps* [54]. These Roadmaps contextualize HCV elimination in that region through previous epidemiologic, implementation science models of care, and qualitative studies completed locally to inform many of the recommendations. However, perhaps more importantly, the *Roadmap* regional core groups underwent extensive consultation across the sector, including with government. To date, both the Ontario [55] and Prairie [56] Roadmaps have been released, both of which include best practices related to Indigenous wellness in the context of HCV. In Ontario for example, over 300 consultations among 130 stakeholders occurred across the sector, resulting in 120 recommendations. An Indigenous working group was formed for the development of the *Roadmap* and is still in place as an Indigenous-led implementation strategy circle for the *Roadmap* in Ontario and a community-directed voice providing insights for policy and practice shifts. Emphasis was placed on “Indigenous Health in Indigenous Hands”, acknowledging unique approaches supporting the distinct and diverse populations of First Nations, Inuit, and Métis people. Nine recommendations were specified, under the following themes: (1) Indigenous -led, whole person care; (2) trauma-informed care and cultural safety in mainstream health services; and (3) addressing rural and remote communities [55]. Importantly, there is also a national Indigenous working group that has not only consulted on the regional Roadmaps, but will develop a specific National Indigenous Roadmap towards HCV elimination [54].

## 9. Data Ownership

The lack of data around infectious diseases is often identified as a gap by researchers, but from an Indigenous perspective, data have additional layers of nuance, including jurisdictional complexities originating in the Indian Act of 1876, and the current oversight of health services by the federal government. Indigenous people are the first scientists of Canada and demonstrate time-honored data practices through connections to the land, observing changes and shifts, and incorporating these observations into wellness practices for the collective benefit of community. However, at present, there is a sense of mistrust in biomedical data practice due to numerous instances of data abuse, data theft, and traditional knowledge being stolen; and the current practice of highlighting the health disparities of Indigenous people without an equal focus on their colonial disruptive origins, ongoing impacts, or the strengths, resilience, and innovative programming led by Indigenous people and organizations. This mistrust is amplified by deceptive research practices, including samples being acquired for one purpose and used for research studies (studies that have ultimately increased stigma and had a disparity focus), and population data collected and disseminated without permission or community involvement, thus furthering harms and misallocation of resources [57].

Reforming assumptions, approaches, and collaboration needs to focus on research originating from an Indigenous community-led desire, in an informed seeking journey that purposefully places Indigenous authority in control, should be an overarching research priority. Learning from Indigenous data governance initiatives and seeking partnerships facilitating incorporation of these frameworks and approaches are key to reflecting on the true purpose of ethical data gathering and knowledge sharing. Academic, funder, organization, and personal reflections on purposeful steps to not cause harm must be at the forefront of all initiatives—it is simply not enough to have good intentions. Parallel consideration should be given to “who” is missing, instead of “what” is missing, in engagement strategies, research partnerships, research proposals, data analysis, and during policy discussion through knowledge dissemination.

Purposeful re-orientation of data governance includes the First Nations OCAP^®^ Principles of Ownership, Control, Access, and Possession [58]. These principles extend beyond the assumptions of data confidentiality to consider community privacy and the impact of data on a wider population. Further enhancing this awareness and creating space is essential within the knowledge economy for Indigenous peoples’ knowledge and data to direct purposeful community benefit. The CARE Principles for Indigenous Data Governance are defined as Collective Benefit, Authority to Control, Responsibility to relationships, and Ethics in representation and participation [59]. Importantly, the Federation of Saskatchewan Indian Nations took these principles and created a cultural responsiveness framework incorporating cultural approaches, self-governance, and data ownership to shape strategic direction, objectives, and an operating plan to a wellness-based approach to transform their health care delivery [60].

The Cedar Project in British Columbia is an example of self-governed data inspiring actionable initiatives [61,62]. This project conducted semi-structured interviews to direct pragmatic recommendations for healing-centered HCV care, resulting in the clearly stated priority for healthcare team members and providers to understand the colonial determinants of current health cycles and the need for purposefully shaping attitudes, spaces, and treatment paths to foster trust, relationality, and circles of supportive care [61,62]. Continuing work with a cohort of Indigenous young people in two British Columbia cities, this project clearly links childhood maltreatment associated with HCV and or HIV infection, leading the project’s Indigenous advisory circle to create trauma-informed programming, healing bundles, connections to culture as protective buffers, and inclusion of young people in program design [62,63]. Using data gathered in respectful, reciprocal, and responsible methods to inspire and direct actionable directives led by Indigenous people creates opportunities for self-governance, healing, and wellness [64,65].

## 10. Conclusions

Structural change is needed in creating a healthcare system with the capacity to provide equitable care and create space for cultural wellness-based perspectives, and liver disease is an urgent health issue. However, to create change, there must be a significant shift in approach. At present, Indigenous people are labeled as target or marginalized populations, with a focus on high prevalence, morbidity, and mortality, but without acknowledging the ongoing devastating impacts of colonization. Yet, with purposeful effort, funding and efforts could be positioned to provide adequate support for Indigenous-led and self-governed healthcare initiatives working within intersectional, non-stigmatizing, harm-reduction, and wellness-based approaches founded in culture. These principles should be embedded into clinical and research training, and all aspects of HCV care. Culture does make a difference, and when the focus is on connection, listening, learning, engaging, and then prioritizing what community members need, and partnering to implement this in program design, the result will be responsive and effective. Strength-based initiatives include family, community, culture, ceremony, and land-based environment perspectives in an anti-colonial approach to healing and wellness, even in the context of HCV.
